# An Artificial Neural Network for Rapid Prediction of the 3D Transient Temperature Fields in Ship Hull Plate Line Heating Forming

**DOI:** 10.3390/ma18215054

**Published:** 2025-11-06

**Authors:** Zhe Yang, Hua Yuan, Zhenshuai Wei, Lichun Chang, Yao Zhao, Jiayi Liu

**Affiliations:** 1 School of Naval Architecture and Ocean Engineering, Huazhong University of Science and Technology, Wuhan 430074, China; 2 Collaborative Innovation Center for Advanced Ship and Deep-Sea Exploration (CISSE), Shanghai 200240, China; 3 Hubei Key Laboratory of Naval Architecture and Ocean Engineering Hydrodynamics (HUST), Wuhan 430074, China

**Keywords:** line heating, 3D transient temperature field, artificial neural network, physics-aware features, near real-time prediction

## Abstract

Line heating processes play a significant role in the fabrication of structural steel components, particularly in industries such as shipbuilding, aerospace, and automotive manufacturing, where dimensional accuracy and minimal defects are critical. Traditional methods, such as the finite element method (FEM) simulations, offer high-fidelity predictions but are hindered by prohibitive computational latency and the need for case-specific re-meshing. This study presents a physics-aware, data-driven neural network that delivers fast, high-fidelity temperature predictions across a broad operating envelope. Each spatiotemporal point is mapped to a one-dimensional feature vector. This vector encodes thermophysical properties, boundary influence factors, heatsource variables, and timing variables. All geometric features are expressed in a path-aligned local coordinate frame, and the inputs are appropriately normalized and nondimensionalized. A lightweight multilayer perceptron (MLP) is trained on FEM-generated induction heating data for steel plates with varying thickness and randomized paths. On a hold-out test set, the model achieves MAE = 0.60 °C, RMSE = 1.27 °C, and R2 = 0.995, with a narrow bootstrapped 99.7% error interval (−0.203 to −0.063 °C). Two independent experiments on an integrated heating and mechanical rolling forming (IHMRF) platform show strong agreement with thermocouple measurements and demonstrate generalization to a plate size not seen during training. Inference is approximately five orders of magnitude (~10^5^) faster than FEM, enabling near-real-time full-field reconstructions or targeted spatiotemporal queries. The approach supports rapid parameter optimization and advances intelligent line heating operations.

## 1. Introduction

Steel plates are widely used structural materials in shipbuilding, aerospace, and automotive manufacturing, and their forming processes remain a central topic in materials engineering. Among these techniques, line heating is not only a widely applied forming method for generating curved geometries but also an essential corrective process for mitigating distortions introduced by subsequent operations such as welding. By applying localized heating along predefined trajectories with flame or induction sources, followed by controlled cooling, line heating induces plastic deformation in specific regions of the plate, enabling the realization of complex target shapes. Advancing the understanding and prediction of this process is therefore crucial for the ongoing digitalization and intelligent transformation of modern manufacturing industries.

The deformation induced during line heating is commonly classified into three mechanisms: temperature gradient, upsetting, and buckling mechanisms [1]. The plate’s internal temperature distribution strongly influences these mechanisms. Accurate regulation of the temperature field is thus indispensable: insufficient control can lead to geometric inaccuracies and even material waste. In addition, industries such as shipbuilding impose strict limits on allowable heating temperatures in order to minimize adverse effects on material microstructures. These considerations collectively highlight the need for a rapid, accurate prediction model of three-dimensional (3D) transient temperature fields during line heating. Such predictive capability would directly contribute to the design of efficient forming strategies, the achievement of target curved geometries, and the preservation of material performance [2,3,4].

Numerous analytical and numerical approaches have been proposed for temperature prediction in line heating. Analytical frameworks rooted in D. Rosenthal’s moving heat source theory [5] achieve computational efficiency by assuming quasi-static conditions. Extensions such as those by Y. Wang et al. [6] provide empirical correlations for isotherm geometry and segment lengths within specific temperature ranges (e.g., 500–800 °C), facilitating engineering applications. However, their reliance on steady-state approximations fundamentally limits their ability to capture the dynamic, time-varying nature of real-world heating and cooling cycles.

In contrast, finite element methods (FEM) have emerged as the dominant tool for high-fidelity simulation. For line heating processes induced by an electromagnetic inductor, Y. Ueda et al. [7,8,9,10] used thermo-elastic-plastic FEM to explore how line heating parameters induce inherent strain due to temperature gradients. Meanwhile, Y. Zhu and Y. Luo [11] developed a fully coupled electromagnetic-thermal-mechanical framework for induction-based line heating, while H. Dong et al. [12] proposed simplified heat source models to reduce computational cost without sacrificing accuracy on flat plates. Despite their predictive reliability, FEM-based approaches suffer from prohibitive computational overhead and lack reusability: any changes in plate thickness, plate geometry, power level, or travel speed necessitate complete model reconstruction and recalculation. This inflexibility severely hinders their integration into iterative, data-driven intelligent manufacturing workflows.

Recently, artificial neural networks (ANN) have shown promise in thermal field prediction across diverse domains. For instance, Physics-Informed Neural Networks (PINNs) have been developed to tackle 2D steady-state heat conduction and convection problems by imposing either hard or soft boundary constraints [13]. To facilitate analysis without labeled data, Physics-Informed Convolutional Neural Networks (PICNNs) have been introduced as surrogate models that learn mappings from heat-source configurations to steady-state temperature distributions [14]. To address transient dynamics, a hybrid approach combining Proper Orthogonal Decomposition (POD) with Convolutional Temporal Networks (CTN) has been proposed to predict temperature fields on aircraft anti-icing surfaces [15]. In the manufacturing sector, models that integrate Graph Neural Networks (GNNs) and Recurrent Neural Networks (RNNs) have been developed for rapid thermal predictions in processes such as additive manufacturing [16,17]. However, these methods remain confined mainly to simplified scenarios: 2D domains, static heat source, surface-only predictions, or quasi-steady assumptions. None adequately addresses the core challenge of line heating: predicting complete 3D transient temperature fields generated by a moving heat source traversing arbitrary paths on metallic plates. This gap critically impedes progress toward real-time intelligent forming systems.

To bridge this gap, this study proposes a novel neural network-based prediction framework for 3D transient temperature fields in annular electromagnetic induction line heating with rectangular cross-section sources. To the best of our knowledge, this integrated framework is proposed here for the first time and is not a direct adaptation from prior work in other fields. Recognizing that thermal gradients are spatially concentrated near the processing path, this method partitions the computational domain into localized sub-regions, thereby reducing learning complexity while retaining essential physical information. Key parameters—including plate thickness, path geometry, input power, and travel speed—are encoded alongside spatial coordinates, time, and dimensionless thermal descriptors within a unified input structure, enabling generalization across varying plate geometries and heating trajectories. A comparatively lightweight Multi-Layer Perceptron (MLP) is employed to capture the dynamics of heat transfer. Training data are generated via FEM simulations under varied thickness, trajectories, and process combinations, balancing accuracy with computational cost.

The remainder of this paper is organized as follows: Section 2 details the methodology, including governing equations, spatial partitioning strategy, data structure design, and neural network architecture. Section 3 presents a comprehensive case study involving plates of varying thickness under randomized processing conditions, covering dataset generation, optimal selection of the partitioning width, data splitting, and hyperparameter tuning. Section 4 introduces the Integrated Heat and Mechanical Roll Forming (IHMRF) system and experimental validation protocol. Section 5 evaluates model performance using training metrics, test-set accuracy, and direct comparisons with experimental measurements, including detailed error analysis. Finally, Section 6 summarizes the conclusions and discusses future research directions.

## 2. Methodology

### 2.1. Governing Equations of Heat Conduction in a Moving Coordinate System

**Figure 1:**
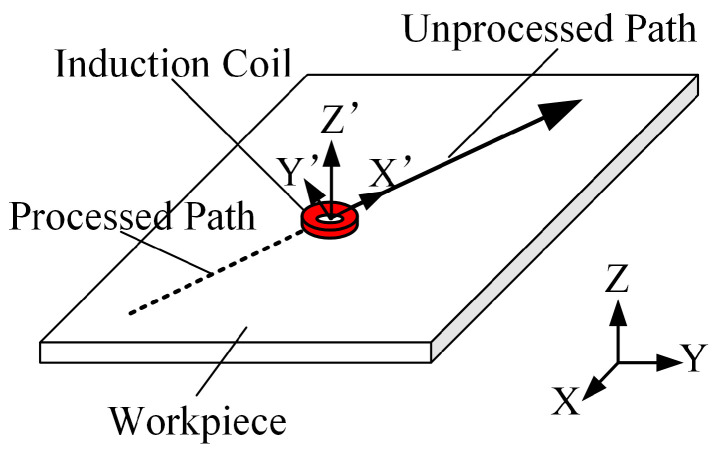
The moving coordinate system used for predicting transient temperature distribution.

To predict the transient temperature distribution inside the workpiece during line heating, a moving coordinate system is established, as illustrated in Figure 1. In this setup, the origin point of the moving coordinate system is at the center of the heat source. The $x^{\prime }$ axis moves along the direction of the heat source at the same speed as the heating source. The $y^{\prime }$-axis is perpendicular to the $x^{\prime }$-axis. The $z^{\prime }$-axis is perpendicular to both the $x^{\prime }$-axis and $y^{\prime }$-axis and follows the right-hand rule. Thus, the relationship between the fixed global coordination system and the moving coordinate system can be expressed easily by Equation (1).

(1) $$\left\{\begin{array}{l} x^{\prime }=x-v\cdot t\\ y^{\prime }=y\\ z^{\prime }=z \end{array}\right.$$

where x, y, and z are the coordinates in the fixed global coordinate system, $x^{\prime }$, $y^{\prime }$, and $z^{\prime }$ are the coordinates in the moving coordinate system, v is the velocity of the heat source and t is the current moment.

The transient temperature field T(x,y,z,t) governing the heat conduction process within the spatial domain Ω of the workpiece can be represented by a three-dimensional transient heat conduction equation, as shown in Equation (2).

(2) $$C_{p}\left(T\right)\rho \left(T\right)\frac{\partial T}{\partial t}=\frac{\partial }{\partial x}\left(k\left(T\right)\frac{\partial T}{\partial x}\right)+\frac{\partial }{\partial y}\left(k\left(T\right)\frac{\partial T}{\partial y}\right)+\frac{\partial }{\partial z}\left(k\left(T\right)\frac{\partial T}{\partial z}\right)+q$$

where $C_{p}(T)$ is the specific heat of the material, ρ(T) is the density of the material, k(T) is the thermal conductivity of the material, and q represents the Joule heat generation density provided by the heat source.

To introduce the relationship between the moving coordinate system and the fixed global coordinate system, the following formulas are derived:

(3) $$\left\{\begin{array}{l} \frac{\partial^{2}T}{\partial x^{2}}=\frac{\partial^{2}T}{\partial {x^{\prime }}^{2}}\\ \frac{\partial^{2}T}{\partial y^{2}}=\frac{\partial^{2}T}{\partial {y^{\prime }}^{2}}\\ \frac{\partial^{2}T}{\partial z^{2}}=\frac{\partial^{2}T}{\partial {z^{\prime }}^{2}} \end{array}\right.$$

(4) $$\frac{\partial T}{\partial t}={\left(\frac{\partial T}{\partial t}\right)}_{M}+\frac{\partial T}{\partial x^{\prime }}\cdot \frac{\partial x^{\prime }}{\partial t}={\left(\frac{\partial T}{\partial t}\right)}_{M}-v\cdot \frac{\partial T}{\partial x^{\prime }}$$

The subscript M in Equation (4) indicates that the term represents the partial derivative with respect to time in the moving coordinate system. Substituting Equations (3) and (4) into Equation (2), the three-dimensional transient heat conduction equation for a moving heat source in the moving coordinate system can be obtained:

(5) $$C_{p}\left(T\right)\rho \left(T\right)\left(\frac{\partial T}{\partial t}-v\frac{\partial T}{\partial x^{\prime }}\right)=k\left(T\right)\left(\frac{\partial^{2}T}{\partial {x^{\prime }}^{2}}+\frac{\partial^{2}T}{\partial {y^{\prime }}^{2}}+\frac{\partial^{2}T}{\partial {z^{\prime }}^{2}}\right)+q$$

The boundary condition is the room temperature at the initial moment, expressed as follows:

(6) $${\mathrm{BC}}_{1}:\;T(x^{\prime },y^{\prime },z^{\prime },0)=T_{env}$$

where the $T_{env}$ is the room temperature.

Heat exchange occurs on all the boundaries (top, bottom, and edges) of the workpiece through convection and radiation processes, which can be represented, respectively, as follows:

$${\mathrm{BC}}_{2}:\;\frac{\partial T}{\partial n}\left|\begin{array}{c} \left(x^{\prime },y^{\prime },z^{\prime },t\right) \end{array}\right.=-\frac{h}{k}\left(T-T_{env}\right)$$

$${\mathrm{BC}}_{3}:\;\frac{\partial T}{\partial n}\left|\begin{array}{c} \left(x^{\prime },y^{\prime },z^{\prime },t\right) \end{array}\right.=-\frac{\epsilon \sigma }{k}\left[{\left(T-T_{Z}\right)}^{4}-(T_{env}-{T_{Z}}^{4})\right]$$

where $\frac{\partial T}{\partial n}$ is the directional derivative of T along the outward unit normal direction n on the boundaries; h is the convection heat transfer coefficient; k is the thermal conductivity of the workpiece; ϵ is the emissivity of the workpiece; σ is the Stefan-Boltzmann constant; $T_{env}$ is the room temperature; and $T_{Z}$ is absolute zero temperature.

### 2.2. Definition and Partitioning of the Critical Thermal Region

To enable efficient and generalizable neural network training, this study introduces a spatial region partitioning strategy that focuses computational attention on the thermally active zone near the moving heat source. This approach is motivated by the observation that plastic deformation and significant temperature gradients are highly localized: regions far from the heat source remain elastic and thermally inert, contributing little to the learning task while increasing data volume unnecessarily.

**Figure 2:**
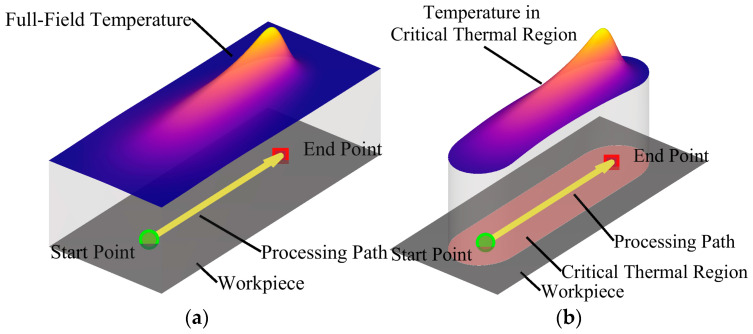
Produce for the partitioning of spatial regions. (**a**) Full transient temperature during heating; (**b**) Extracted localized region used for neural network training.

As illustrated in Figure 2, a capsule-shaped domain is adopted to enclose the critical thermal region. The capsules’ longitudinal axis aligns with the path traced by the moving heat source, connecting its start and end points. Its thickness matches the workpiece, while its lateral width $l_{\mathrm{ctr}}$ is adjustable to capture sufficient thermal influence. All subsequent analysis and training are restricted to this bounded subdomain.

The choice of $l_{\mathrm{ctr}}$ involves balancing model accuracy and computational efficiency. An overly large $l_{\mathrm{ctr}}$ includes redundant low-gradient regions, inflating training data size and resource demands. In contrast, an overly small $l_{\mathrm{ctr}}$ excludes boundary-adjacent thermal features, impairing the model’s ability to generalize near domain edges. Additionally, optimal $l_{\mathrm{ctr}}$ depends on heat source geometry and process parameters. While systematic optimization of $l_{\mathrm{ctr}}$ is reserved for future work, Section 3 demonstrates a practical selection based on empirical validation.

**Figure 3:**
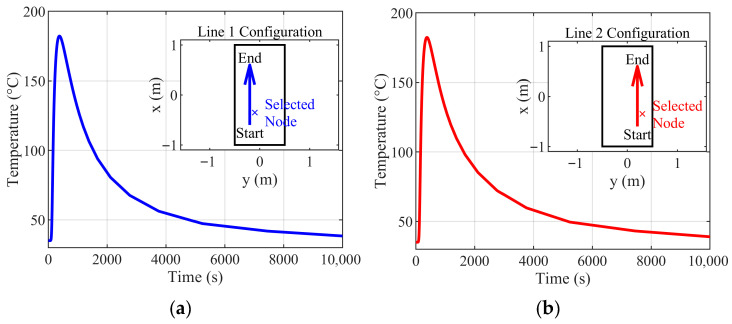
Consistency in temperature at matched relative locations within the partitioned region. (**a**) Path 1; (**b**) Path 2.

The effectiveness of this spatial partitioning is confirmed by examining temperature similarity across different heating paths within the defined region. As shown in Figure 3, two distinct line heating trajectories (Path 1 and Path 2) applied to a 2 m × 1 m × 0.02 m plate—with identical parameters except for location—produce nearly identical temperature histories at corresponding relative positions (e.g., 0.1 m perpendicular offset, 0.25 m along the path). Minor deviations occur only during cooling, indicating substantial local thermal similarity.

This observed invariance justifies the use of a single neural network architecture to predict transient temperature fields across diverse processing paths—demonstrating that spatial partitioning not only reduces computational load but also enhances model generalizability by focusing learning on physically relevant features.

### 2.3. Data Structure for Training Neural Network

**Figure 4:**
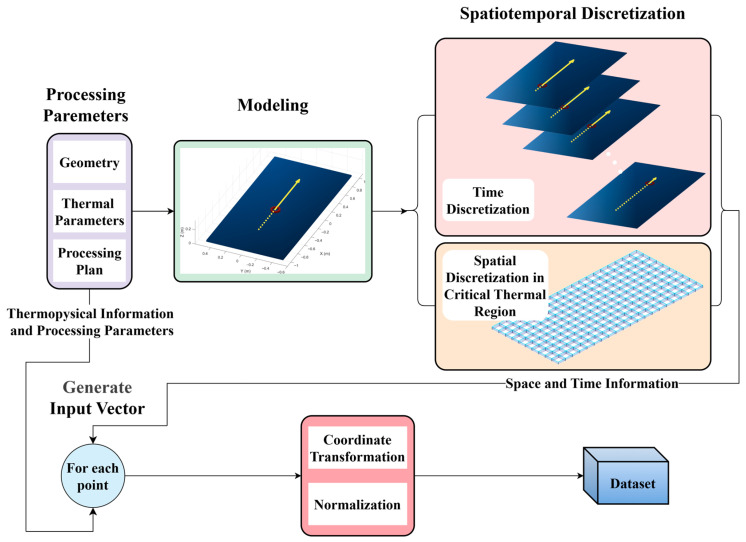
Workflow for dataset construction.

Figure 4 illustrates the data-generation workflow. To remove size effects caused by variable sample dimensions (e.g., path or plate) during training, each spatiotemporal sample is reorganized into a fixed-length, one-dimensional feature vector. While this operation inherently disrupts native spatiotemporal correlations, it significantly enhances computational flexibility—enabling point-wise temperature prediction without full-field computation. To compensate for lost structural context, each vector is enriched with physically meaningful descriptors that preserve essential boundary, material, heat source status, and time information.

For each processing configuration, the computational domain is instantiated, and both space and time are discretized. At every spatiotemporal location, thermophysical and processing attributes are assembled to form an input vector. The global coordinates are then mapped to a local, path-aligned coordinate system, followed by normalization. The resulting vectors are aggregated to build the training dataset. (Figure 4).

#### 2.3.1. Composition of the Input Vector

**Figure 5:**
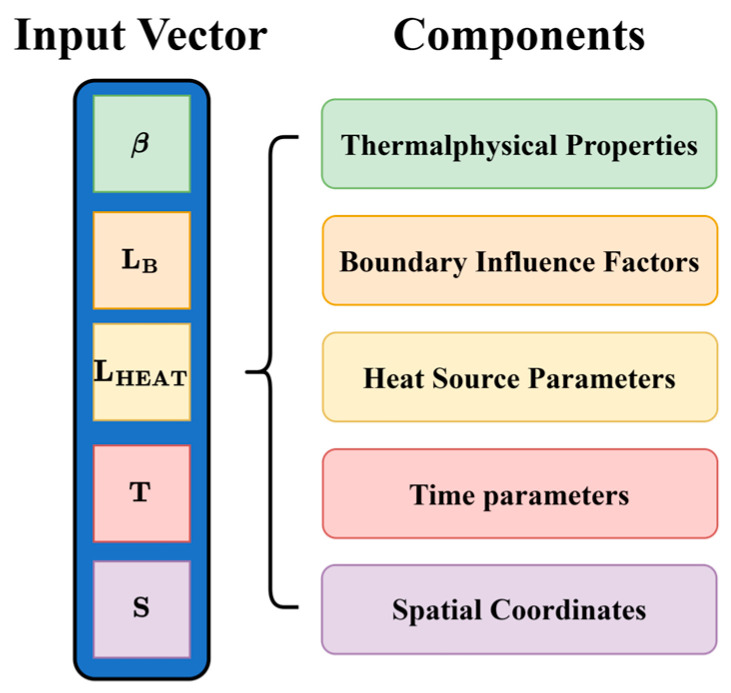
Composition of the Input Vector.

As shown in Figure 5, each input vector contains five groups of features: thermophysical properties, boundary influence factors, heat source parameters, time parameters, and spatial coordinates.

Thermophysical Properties β. Following Wei et al. [18], two dimensionless groups (Equations (7) and (8)) are included to mitigate dimensional disparity during line heating. Here, k is the thermal conductivity of the workpiece, $C_{p}$ is the specific heat capacity of the material, d is the thickness of the plate, v is the travel speed of the heat source, Q is the heat source input power, and $T_{\max}$ is the peak plate temperature. In addition, three processing parameters—plate thickness d, source speed v, and maximum processing temperature $T_{\max}$—are included.

(7) $$\pi_{T1}=\frac{k}{C_{p}dv}$$

(8) $$\pi_{T2}=\frac{Q}{C_{p}vd^{2}T_{\max}}$$

Boundary Influence Factors $L_{B}$. To compensate for boundary information loss induced by one-dimensional vectorization, six scalars are used to describe the influence of six faces of a cuboidal workpiece under a specific thermal boundary condition (e.g., convection or radiation). To reduce collinearity among features and prevent misassociation during backpropagation, the four in-plane factors ($L_{1}-L_{4}$) are obtained via a nonlinear mapping of the Euclidean distances as in Equation (9). For the two out-of-plane factors ($L_{5}-L_{6}$), the Euclidean distances to the top and bottom surfaces are used directly.

(9) $$L_{i}=e^{-8l_{i}}$$

where $l_{i}$ is the distance to the i-th lateral face and $L_{i}$ is the characteristic length scale.

Heat Source Parameters $L_{HEAT}$. Two values are included: the total travel length of the heat source, $l_{h}$, and the travel distance at time t, $l_{ht}(t)$.

Time Parameters T. Three temporal features encode process dynamics: the total heating duration $t_{heat}$, the current time t, and a binary activation flag $F_{heat}$ indicating whether the heat source is active at time t.

Spatial Coordinates S. The x and y coordinates are used in their raw form, while the vertical coordinate is normalized according to Equation (10) to obtain $\bar{z}$. The relative position to the heat source is represented by the distance $l_{pht}$ from the source center to the point and the cosine of the angle cosθ between the source’s direction of advance and the vector pointing from the source to the point.

(10) $$\bar{z}\;=\frac{z}{d}$$

where z is the z-coordinate of the spatial point, d is the thickness of the workpiece, and $\bar{z}$ is the normalized vertical coordinate.

#### 2.3.2. Coordinate Transformation and Normalization

A local coordinate system is established for each simulation case, anchored at the heat source’s starting position. The x− axis aligns with the heat source’s motion direction; the y− axes lies perpendicular within the horizontal plane; and the z− axis completes the right-handed orthogonal system vertically.

Global-to-local transformation involves computing a 2D affine operator that combines a translation (T) and a rotation (R) matrix, tailored to each processing path. All spatial components in the input vector are reprojected accordingly to enhance cross-case transferability.

After transformation, features are normalized as follows:

1. 1. Dimensionless groups are scaled using Equation (11).

(11) $$\beta_{Norm}=\frac{\log\left(\beta \right)-\log\left(\beta_{\min}\right)}{\log\left(\beta_{\max}\right)-\log\left(\beta_{\min}\right)}$$

where $\beta_{\max}$ and $\beta_{\min}$ denote the dataset-wide extrema of the corresponding quantity β.

2. Time-related features are normalized via Equation (12).

(12) $$t_{Norm}=\frac{\log\left(t+1\right)-\log\left(t_{\min}+1\right)}{\log\left(t_{\max}+1\right)-\log\left(t_{\min}+1\right)}$$

where $t_{\max}$ and $t_{\min}$ are the extrema of time t.

3. Length-, velocity-, and temperature-related quantities are normalized according to Equation (13).

(13) $$l_{Norm}=\frac{l-l_{\min}}{l_{\max}-l_{\min}}$$

where $l_{\max}$ and $l_{\min}$ are the maximum and minimum values of the associated parameters l, respectively.

### 2.4. The Architecture of the Neural Network

**Figure 6:**
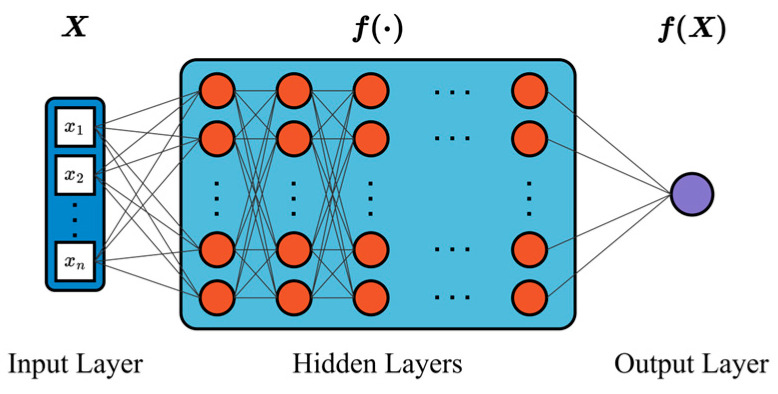
Architecture of the multilayer perceptron.

As shown in Figure 6, a Multilayer Perceptron (MLP), i.e., a feedforward neural network trained via backpropagation, is adopted in this work. The model is treated as a parametric mapping: $f:{\mathbb{R}}^{n}\to {\mathbb{R}}^{m}$, $\hat{T}=f(X)$. Here, n is the input vector dimension and m is the output dimension; in this study, m=1, corresponding to the temperature $\hat{T}$ at a spatial point $\left(x_{i},y_{i},z_{i}\right)$ and time t. Training seeks to learn heat-conduction patterns by minimizing the loss $L(\hat{T},T)$ over the dataset, with the pointwise discrepancy measured by the Huber loss (Equation (14)) between prediction $\hat{T}$ and target T.

The architecture comprises an input layer, a stack of hidden layers with uniform width, and a single-neuron output layer producing $\hat{T}(x_{i},y_{i},z_{i},t)$. The input-layer size matches the feature dimension defined in Section 2.3. The number of hidden layers and their width are selected based on the learning scope of the critical thermal region in the processing-parameter space; a representative configuration is reported in Section 3.4.

Leaky ReLU is used as the activation across all layers (input-hidden, hidden-hidden, hidden-output). Weights and biases are initialized using the Kaiming uniform scheme, and the Adam optimizer is employed to update parameters during training. To balance robustness and convergence speed, Huber Loss is adopted as the training criterion (Equation (14)).

(14) $$L\left(\hat{T},T\right)=\left\{\begin{array}{c} \frac{1}{2}{\left(T-\hat{T}\right)}^{2},\;\;\;if\left|T-\hat{T}\right|\leq \delta ,\\ \delta \cdot \left(\left|T-\hat{T}\right|-\frac{1}{2}\delta \right),\;\;\;\;otherwise \end{array}\right.$$

where T is the actual value, $\hat{T}$ is the neural network’s predicted value, and δ is the threshold of the Huber function.

## 3. Case Study: Line Heating with Random Processing Paths and Varying Plate Thickness

This section investigates training the framework for line heating on plates of varying thickness with randomly sampled processing parameters. An electromagnetic induction coil generates a moving heat source on rectangular steel plates measuring 2 m × 1 m with thickness of 0.012, 0.016, and 0.020 m. This pipeline covers FEM-based data generation, proper $l_{ctr}$ selection, boundary factor design, dataset partitioning, and hyperparameter optimization.

### 3.1. Generation of Sample Data

To balance accuracy and efficiency, the training data were generated using FEM. For direct comparison with experiments, a rectangular cross-section annular induction coil is adopted, consistent with the Integrated Heating and Mechanical Rolling Forming (IHMRF) equipment introduced in Section 4.

Due to the skin effect in high-frequency induction, resistive heating concentrates near the plate surfaces; thus, a surface heat flux serves as an effective surrogate. For an annular inductor, the equivalent heat source can be described by a Gaussian function (Equation (15)):

(15) $$q\left(r\right)=q_{m}e^{-C{\left(r-R_{0}\right)}^{2}}$$

where $q\left(r\right)$ is the heat flux at radial position r, $q_{m}$ is the peak flux, C is the heat concentration coefficient, $R_{0}$ is the radial position of the peak heat flux $q_{m}$ occurs. Integrating the surface heat flux over the entire spatial domain yields the input power Q:

(16) $$Q=\int_{0}^{2\pi }d\theta \int_{0}^{+\infty }q\left(r\right)rdr$$

Substituting Equation (15) into (16) gives the relation between the peak flux $q_{m}$ and the input power Q:

(17) $$q_{m}=\frac{Q}{\pi R_{0}\sqrt{\frac{\pi }{C}}+\frac{\pi }{C}e^{-C{R_{0}}^{2}}}$$

Finally, substituting Equation (17) into (15) provides the complete source expression.

(18) $$q\left(r\right)=\frac{Q}{\pi R_{0}\sqrt{\frac{\pi }{C}}+\frac{\pi }{C}e^{-CR_{0}^{2}}}e^{-C{\left(r-R_{0}\right)}^{2}}$$

FEM simulations are conducted in ABAQUS 2023 using four-node quadrilateral shell elements. As in Section 2.1, the equivalent heat source is implemented via a user subroutine using Equation (18). Following Wei et al. [19], A 10 mm × 10 mm mesh achieves a suitabletrade-off between accuracy and efficiency. The final model contains 20,000 elements and 20,301 nodes.

The parameter ranges for this study were selected to reflect practical industrial applications and are detailed in Table 1. The maximum heating temperature is constrained to 400–800 °C, in accordance with the Shipbuilding and Repair Quality Standards [20]. The selected ranges for plate thicknesses represent typical dimensions used in shipbuilding, while the heat source velocities correspond to standard processing conditions. Q235 structural steel is employed in this study, with its thermal properties detailed in Table 2 [21]. A coordinate system is established with the origin at the plate’s geometric center, the *x*-axis along the long edge, and the *y*-axis along the short edge. Starting and ending points are uniformly sampled within the plate boundaries, specified as x ∈ [−1, 1] m and y ∈ [−0.5, 0.5] m. For each plate thickness, 90 distinct line-heating cases are generated, resulting in a total of 270 transient temperature-field datasets.

### 3.2. Selection of Critical Thermal Region Width

Vertical deflection—a critical metric in forming—is used to determine optimal critical thermal region width ($l_{ctr}$). An extreme scenario is examined in which the field is truncated at the capsule boundary and the exterior is held at ambient temperature. Because compressive plastic strain arises from constrained thermal expansion adjacent to unheated regions, placing the heating line at the plate center provides a conservative and representative condition.

**Figure 7:**
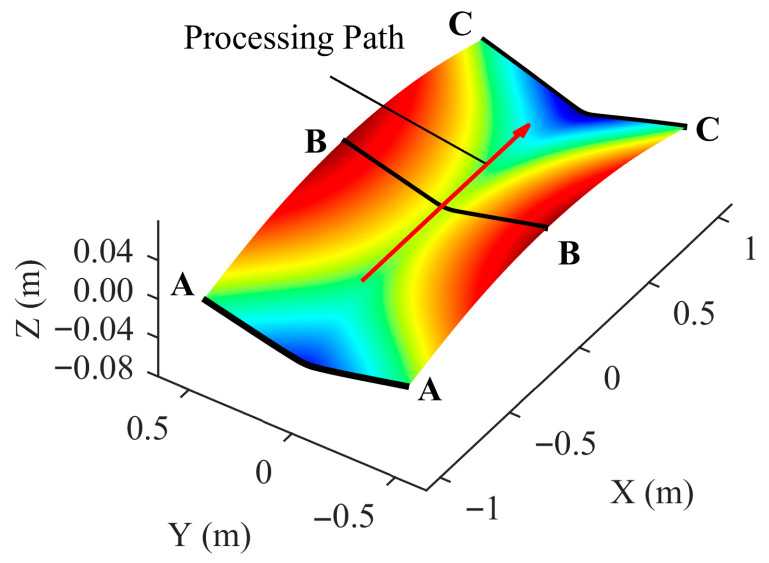
Selection of processing path and reference sections. Note that the *z*-axis is vertically exaggerated by a factor of 5 for visualization clarity.

At a fixed maximum temperature of 800 °C, all combinations of speed and thickness in Table 1 are tested. As shown in Figure 7, deviations in vertical deflection are evaluated for several critical thermal region widths using sections A-A, B-B, and C-C. Table 3 reports the maximum absolute and relative errors for each width. Given that on-site deflection measurements typically accept errors of up to 1 mm, a critical thermal region width of 0.3 m is selected, which limits the maximum absolute error to less than 1 mm and the maximum relative error to below 8%, while still retaining information near the boundary and limiting dataset size.

### 3.3. Dataset Generation and Partitioning

In the line heating process of this case study, only natural convection and thermal radiation act at the thermal boundaries. As these processes coexist throughout, a single set of six boundary influence factors suffices to encode their combined effect in the critical thermal region. Consequently, the input-vector dimension—and the MLP input-layer size—is 21. Samples are generated as described in Section 2.3

The dataset is split into non-overlapping training (90%), validation (5%), and test (5%) sets.

For each line-heating case:

1. Transient temperature data are jointly filtered by vertical coordinate z and time t to form whole 2D slices. Further, 5% of these complete slices are assigned to the test set; the remaining 95% are reserved for subsequent partitioning.
2. The remaining data are randomly shuffled; 5% are sampled by index as the validation set, and the rest constitute the training set.

This strategy ensures that the test set comprises complete 2D temperature fields, facilitating qualitative and quantitative assessment of predicted spatial distributions. Table 4 reports the number of input vectors in each subset.

### 3.4. Optimization of Hyperparameters

In this case study, hyperparameters are tuned using Optuna 4.2.1 [22], including the number of hidden layers, the number of neurons per hidden layer, the Huber loss threshold, and the learning rate; the search ranges are listed in Table 5. Fifty pruning-enabled trails are performed using samples from Section 3.3. The optimal configuration is summarized in Table 6.

## 4. Experimental Validation Procedure

This section outlines the Integrated Heating and Mechanical Rolling Forming (IHMRF) system developed at Huazhong University of Science and Technology (HUST). It presents two pure line heating experiments conducted on this platform to validate the framework.

**Figure 8:**
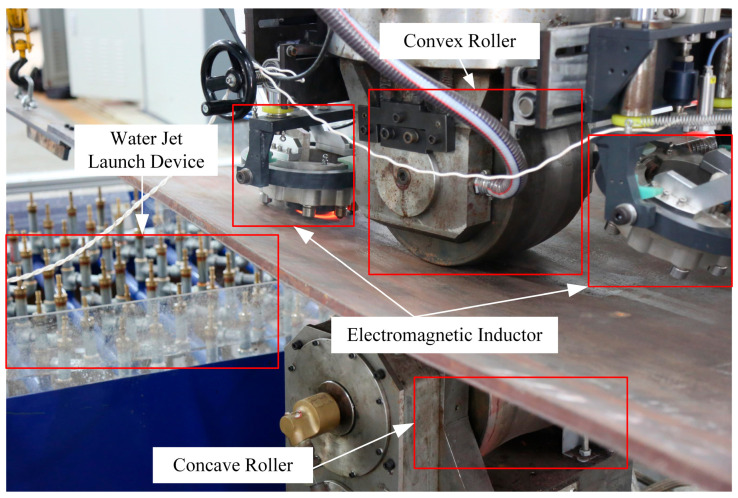
Photograph of the IHMRF Experimental Setup.

Figure 8 shows the IHMRF setup, an automated forming system for shipbuilding double-curved plates capable of applying line heating and mechanical pressing loads simultaneously [23]. For the present validation, only line heating is activated. The heating subsystem employs an electromagnetic induction unit with a maximum power of 160 kW as the heat source. A rectangular cross-section annular coil with a 100 mm diameter is mounted on an adaptive positioning device, which maintains a constant 5 mm standoff from the plate surface [24]. Four motors suspend the workpiece, each connected to a lifting ring welded to a corners; the motors continuously regulate posture to keep the plate parallel to the ground. During pure line heating, the upper and lower rollers exert minimal force, sufficient for feed motion but insufficient to induce plastic deformation by pressing. Temperatures are recorded using thermocouples welded to the plate. Sensors are placed near the processing path, avoiding interference from the rollers to ensure representative measurements.

Two experiments were performed to assess model performance under practical conditions. Experiment 1 validates the engineering applicability and temperature-field accuracy of the method on a 2 m × 1 m × 0.12 m Q235 plate. Experiment 2 evaluates adaptability to a different size (1 m × 1 m × 0.02 m), which was not included in the training.

### 4.1. Experiment 1: Engineering Validation of the Model

**Figure 9:**
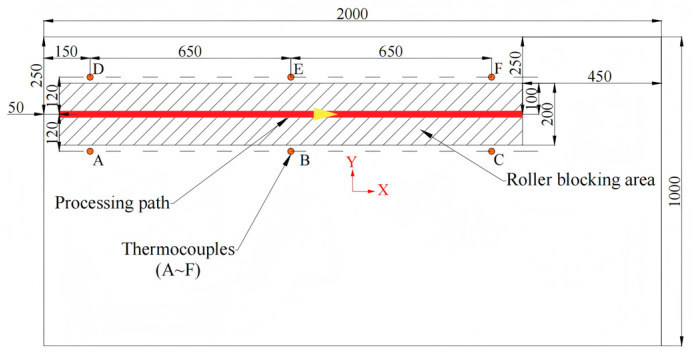
Layout of processing line and thermocouple locations for Experiment 1. The yellow arrows along the machining path indicate the direction of machining.

**Figure 10:**
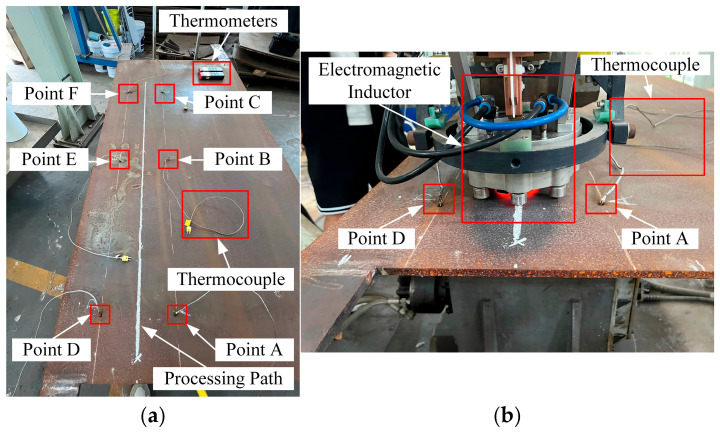
Experiment 1: Photographs. (**a**) Layout of the Thermocouples; (**b**) Photograph of the Heating Phase.

Experiment 1 involves pure line heating of a 2 m × 1 m × 0.12 m rectangular Q235 plate. The objective is to confirm the model’s suitability for engineering processing conditions and its temperature-prediction accuracy. The processing parameters are listed in Table 7. All values lie within the range of Table 1, but the specific combination does not duplicate any of the 270 training cases. The processing path and thermocouple locations are shown in Figure 9, where a coordinate system is established with its origin at the plate’s center. Representative operation photographs are provided in Figure 10.

### 4.2. Experiment 2: Adaptability to Different-Sized Plates

**Figure 11:**
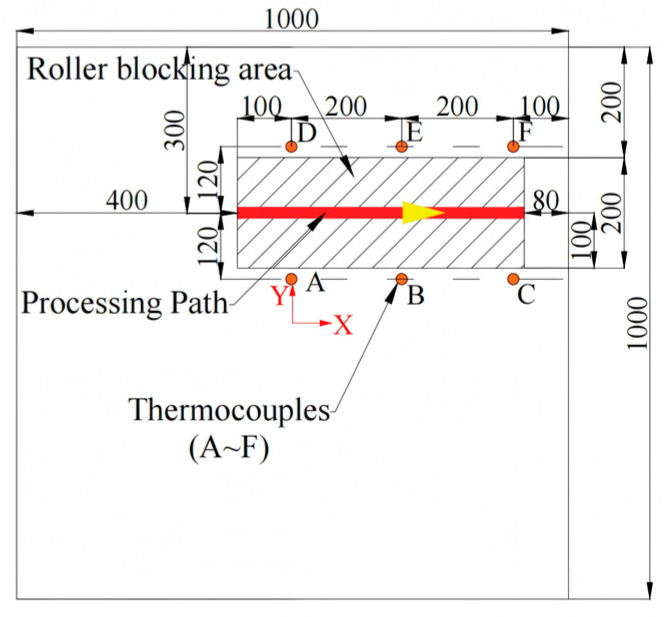
Layout of processing lines and thermocouple locations for Experiment 2. The yellow arrows along the machining path indicate the direction of machining.

**Figure 12:**
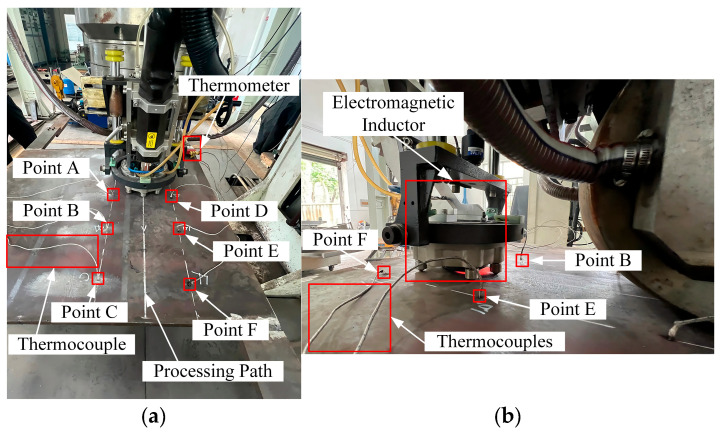
Experiment 2: Photographs. (**a**) Layout of the Thermocouples; (**b**) Photograph of the Heating Phase.

Experiment 2 consists of pure line heating of a 1 m × 1 m × 0.02 m Q235 plate to examine model performance on a plate size not seen during training. The processing parameters are given in Table 8. The path layout and thermocouple are shown in Figure 11, where a coordinate system is established with its origin at the plate’s center. Photographs of the heating operation are presented in Figure 12.

## 5. Results and Discussion

This section defines the evaluation metrics, reports performance on the test set, and compares model predictions with experimental measurements.

### 5.1. Metrics

Referring to the study of J. Chen et al. [25], six metrics are used to assess predictive performance: Mean Absolute Error (MAE), Mean Absolute Percentage Error (MAPE), Root Mean Square Error (RMSE), correlation of coefficient ($R^{2}$), Mean of Median Absolute Error (MedAE), and Mean of Maximum Absolute Error (MMaxAE). A 99.7% error interval is additionally estimated via a bootstrap procedure with 100,000 subsamples and 10,000 iterations. The formal definitions are given in Equations (19)–(24).

(19) $$MAE=\frac{1}{M}\sum_{j=1}^{M}\left(\frac{1}{N}\sum_{i=1}^{N}\left(\left|T_{i,j,predict}-T_{i,j,true}\right|\right)\right)$$

(20) $$MAPE=\frac{1}{M}\sum_{j=1}^{M}\left(\frac{1}{N}\sum_{i=1}^{N}\left(\left|\frac{T_{i,j,predict}-T_{i,j,true}}{T_{i,j,true}}\right|\right)\right)\times 100\%$$

(21) $$RMSE=\frac{1}{M}\sum_{j=1}^{M}\sqrt{\frac{1}{N}\sum_{i=1}^{N}{\left(T_{i,j,predict}-T_{i,j,true}\right)}^{2}}$$

(22) $$R^{2}=\frac{1}{M}\sum_{j=1}^{M}\left(1-\left(\frac{\sum_{i=1}^{N}{\left(T_{i,j,predict}-T_{i,j,true}\right)}^{2}}{\sum_{i=1}^{N}{\left(T_{i,j,t\mathrm{rue}}-\frac{1}{N}\sum_{i=1}^{N}T_{i,j,true}\right)}^{2}}\right)\right)$$

(23) $$MedAE=\frac{1}{N}\sum_{j=1}^{N}\left(\mathrm{median}\left(\left|T_{i,j,predict}-T_{i,j,true}\right|\right)\right)\;i=1,2,3,\ldots ,N$$

(24) $$MMaxE=\frac{1}{M}\sum_{j=1}^{M}\left(\max\left(\left|T_{i,j,predict}-T_{i,j,true}\right|\right)\right)\;i=1,2,3,\ldots ,N$$

In these expressions, $T_{i,predict}$ and $T_{i,true}$ denote the predicted and reference temperatures at node i for case j; N is the number of nodes in case j, and M is the number of test cases.

Taken together, these metrics provide a comprehensive view of accuracy and error dispersion: MAE and RMSE quantify average and quadratic deviations; MAPE and $R^{2}$ evaluate relative error and goodness-of-fit; MedAE captures typical error levels; MMaxAE reflects worst-case deviations; and the 99.7% interval characterizes the primary spread of residuals.

### 5.2. Results on Test Set

**Figure 13:**
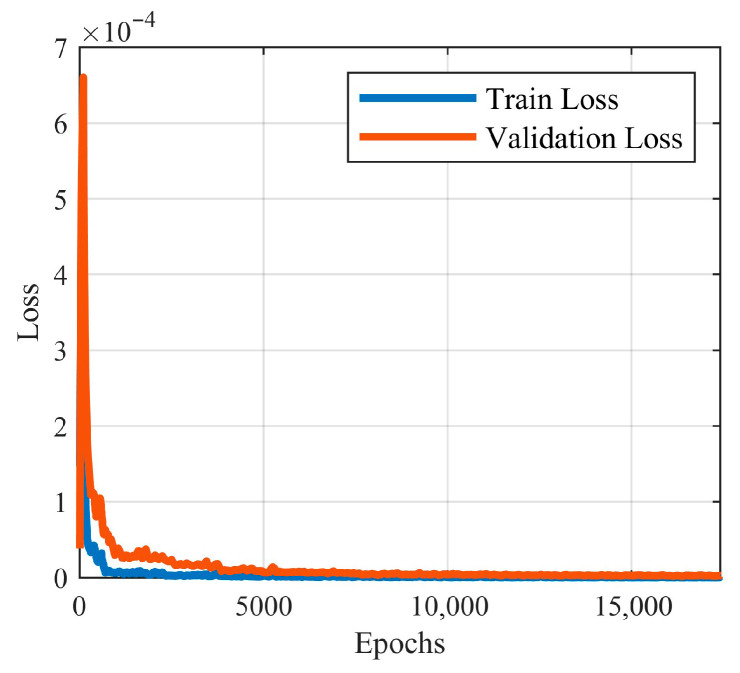
Variation of Huber loss curve over epochs.

Training was performed in PyTorch 2.5.1 on an NVIDIA RTX 4080 Super GPU manufactured by Galaxy in Shenzhen, China, with a budget of 20,000 epochs and early stopping enabled. The best model was achieved at epoch 17,364 after approximately 236 h; during inference, it operates independently of FEM. Figure 13 shows the evolution of the Huber loss over epochs.

#### 5.2.1. Qualitative Assessment of Temperature Fields

**Figure 14:**
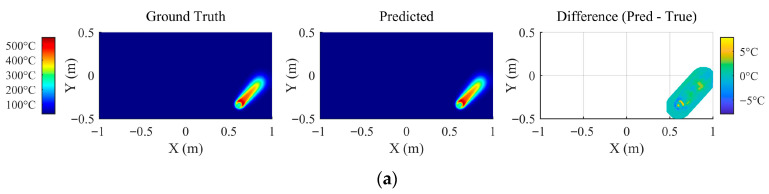

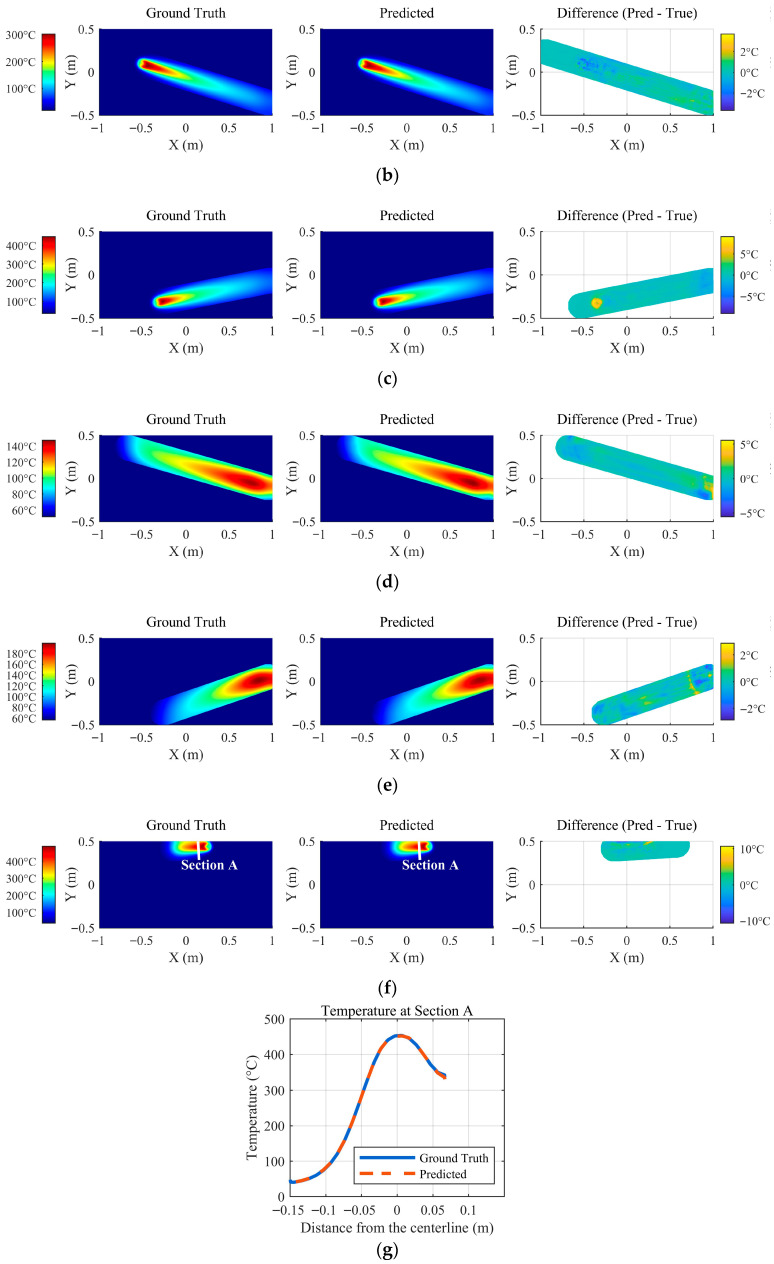
Ground truth, predicted values, and deviations for sliced 2D temperature fields under different processing conditions. (**a**–**c**) Heating Stage; (**d**,**e**) Cooling Stage; (**f**,**g**) Edge-Proximity Heating.

Figure 14 presents a qualitative comparison between the model’s predictions and the FEM ground truth for six representative cases, covering heating, cooling, and near-boundary processing paths. Visually, the predicted temperature fields show excellent agreement with the ground truth, accurately capturing the temperature distribution patterns learned from the sample data.

Heating Stage: The model robustly captures the characteristic asymmetrical temperature distribution induced by the moving heat source, even under varying peak temperatures and speeds. While minor deviations are concentrated in the region of high nonlinearity and steep gradients near the heat source, their magnitude is minimal and well within acceptable limits for practical applications.

Cooling Stage: During cooling, the model successfully predicts the distortion of the temperature field caused by the “edge effect”—the differential heat transfer at the plate periphery. This result corroborates the effectiveness of the boundary-influence factors included in the model’s input vector. The prediction errors in this stage are uniformly distributed, with slightly larger deviations near the boundaries, and remain small.

Edge-Proximity Heating: When the heating line is near a plate edge, the model accurately reproduces the pronounced distortion and asymmetry in the temperature field. The absence of significant asymmetric error along the path further validates the crucial role of boundary-influence factors in accurately capturing the intricate heat transfer processes affected by geometric effects.

#### 5.2.2. Quantitative Evaluation

The model’s predictive performance was further validated through a quantitative analysis of the test set, with key metrics detailed in Table 9. The model demonstrates exceptional predictive accuracy, as indicated by low MAE and RMSE, reflecting minimal overall residuals. Additionally, a very low MAPE and a high coefficient of determination (R^2^ = 0.995) underscore the precision of the predictions. The narrow 99.7% error interval (−0.2031 °C to −0.0627 °C) reveals that errors are closely clustered with a slight negative bias. While occasional outliers appear, primarily near the boundaries of the input space, they remain acceptable for engineering applications. These quantitative results confirm the high-fidelity predictive capability of the trained model.

### 5.3. Comparison with Experimental Data

**Figure 15:**
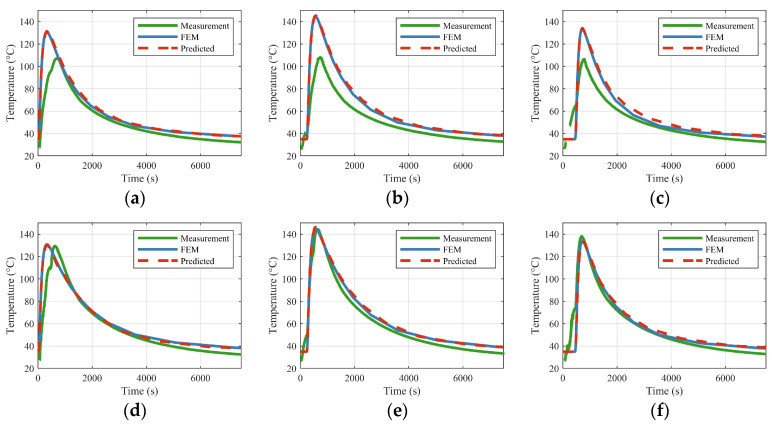
Comparison of measurements, model predictions, and FEM results for points A–F in Experiment 1. The figures designated as (**a**–**f**) illustrate the measured, modeled, and finite element method (FEM) time-dependent evolution at locations A through F, respectively.

**Figure 16:**
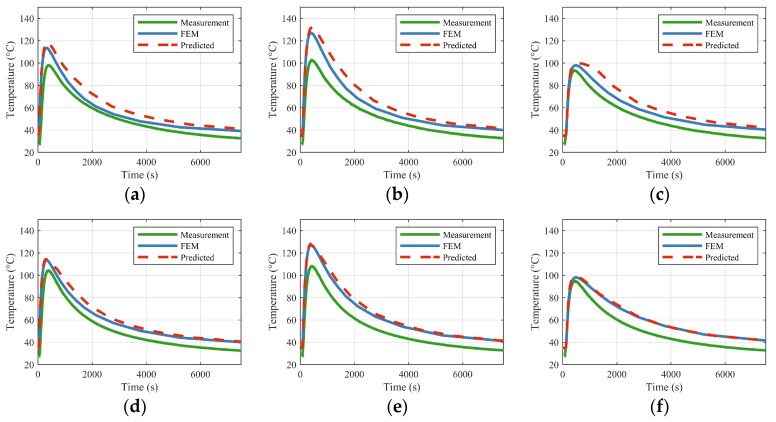
Comparison of measurements, model predictions, and FEM results for points A–F in Experiment 2. The figures designated as (**a**–**f**) illustrate the measured, modeled, and finite element method (FEM) time-dependent evolution at locations A through F, respectively.

To assess the model’s validity against physical reality, its predictions were compared with thermocouple measurements from two experiments (Exp. 1 and Exp. 2), along with their corresponding FEM simulations. As illustrated in Figure 15 and Figure 16, the temperatures predicted by the trained model align closely with both the experimental data and the FEM results. The model effectively captures the overall thermal history, including the crucial transition between heating and cooling phases. The excellent concordance between the model’s predictions and the FEM data confirms that the model has successfully learned the underlying physical principles—such as the effects of thermophysical parameters, plate properties, and boundary conditions—directly from the training data.

A primary objective of Experiment 2 was to evaluate the model’s generalization capabilities. The model, having been trained exclusively on data from 2 m × 1 m plates, demonstrated its ability to accurately predict the transient temperature field for a 1 m × 1 m plate. This precise inference of an unseen geometry, achieved without remeshing or resolving, underscores a significant advantage of the proposed framework over traditional FEM: its case-agnostic predictive power.

Although the overall agreement is significant, minor discrepancies are noted between the model/FEM predictions and the experimental measurements. These deviations primarily result from the physical deflection of the workpiece under the four-point lifting scheme used in the experiments. The sag induced by gravity affects the air gap between the induction coil and the plate, consequently altering the magnetic flux distribution and the effective heat input. This phenomenon is more pronounced in thinner, larger plates and is especially evident in the central regions. This physical explanation aligns with the error patterns observed in Figure 15 and Figure 16, where centrally located points (Points A–C) exhibit greater deviations compared to those adjacent to the edges (Points D–F).

### 5.4. Computational Efficiency

Beyond predictive accuracy, the proposed framework delivers a transformative advantage in computational speed. As summarized in Table 10, the end-to-end prediction time, including file I/O, is approximately five orders of magnitude (~10,000×) faster than the conventional FEM analysis for both experiments. The significant reduction in computation time enables near-real-time assessment of temperature histories in proximity to the processing path. This capability, which is not achievable through conventional simulation methods, is essential for practical applications such as process control.

## 6. Conclusions

This work proposes a neural network-based framework for the rapid prediction of 3D transient temperature fields in line heating. Its effectiveness was validated on a large hold-out test set and through two independent experiments conducted on the IHMRF platform. The main conclusions are as follows:

1. This study introduces a unified, data-driven framework that efficiently predicts the whole 3D transient temperature field over a complete line heating cycle. By localizing the predictive domain to a critical thermal region capsule and embedding thermophysical properties, boundary influence factors, and path-aligned coordinates, the method captures key process physics while alleviating the training challenges of large spatiotemporal domains. The resulting model generalizes across plate geometries, thickness, and processing parameters.
2. The proposed framework achieves substantial gains in computational efficiency with comparable accuracy to FEM solutions. The model supports versatile inference—returning either full-field results or pointwise queries—and delivers predictions approximately five orders of magnitude faster than FEM under equivalent output conditions, while maintaining high fidelity across standard error metrics.
3. Two pure line heating experiments on the IHMRF system verify engineering applicability and cross-size generalization. Notably, the model trained on 2 m × 1 m plates accurately predicts the transient temperature field for a 1 m × 1 m plate without remeshing or resolving, demonstrating strong out-of-sample adaptability.

Overall, the proposed approach enables fast, high-fidelity temperature prediction suitable for in situ parameter tuning, rapid what-if evaluation, and near-real-time support for automated line heating operations.

A current limitation is that all training data were generated via FEM, which may transfer modeling approximations into the learned model. Future work will integrate experimentally measured data with computational data to reduce modeling bias, extend the framework to multiple heat sources and more complex boundary conditions, and explore curved plates, and investigate the influence of key parameters such as plate thickness and material properties. Additional directions include incorporating physics-informed regularization and quantifying predictive uncertainty for robust deployment.

## Figures and Tables

**Table 1:** Overview of processing parameters ranges.

Maximum Processing Temperature	Heat Source Speed	Plate Thickness	Start/End Coordinates of Processing Path
400~800 °C	1 mm/s	12 mm	Random select from [−1, 1] in *x*-axis and [−0.5, 0.5] in *y*-axis
	2 mm/s		
	3 mm/s	16 mm	
	4 mm/s		
	5 mm/s	20 mm	
	6 mm/s		

**Table 2:** Thermal properties of Q235 structural steel.

Temperature (°C)	Thermal Conductivity (W∙(mm∙°C)^−1^)	Specific Heat (KJ∙(kg∙°C)^−1^)
0	51.9 × 10^−3^	486
100	51.1 × 10^−3^	486
200	48.6 × 10^−3^	498
300	44.4 × 10^−3^	515
400	42.7 × 10^−3^	536
500	39.4 × 10^−3^	557
600	35.6 × 10^−3^	586
700	31.8 × 10^−3^	619
800	26.0 × 10^−3^	691
900	26.4 × 10^−3^	695

**Table 3:** Maximum absolute and relative errors of vertical deflection for different widths of critical thermal region.

$l_{ctr}$	Maximum Absolute Error	Maximum Relative Error
0.25 m	1.354 mm	8.08%
0.30 m	0.693 mm	6.55%
0.35 m	0.521 mm	4.92%
0.40 m	0.348 mm	3.28%
0.45 m	0.250 mm	2.36%

**Table 4:** Number of input vectors across training, validation, and test sets.

	Number of Input Vector
Training Set	1,692,643,142
Validation Set	88,875,220
Test Set	93,402,033

**Table 5:** Ranges of hyperparameter values.

The Number of Hidden Layer	The Number of Nodes per Payer	The Huber Function Threshold	The Learning Rate
$N_{layers}$	$N_{nodes}$	δ	lr
6~10	64~256	0.1~20	$1\times 10^{-2}$ $\sim 1\times 10^{-5}$

**Table 6:** Optimal hyperparameter configurations.

The Number of Hidden Layer	The Number of Neurons per Payer	The Huber Function Threshold	The Learning Rate
$N_{layers}$	$N_{nodes}$	δ	lr
10	146	0.4965	$4.92\times 10^{-4}$

**Table 7:** Processing parameters for line heating in Experiment 1.

Parameters	Start Point	End Point	Power	Velocity of Heat Source	Maximum Processing Temperature
Unit	m	m	W	mm/s	°C
Value	(−0.95, 0.25)	(0.55, 0.25)	26,927	3	800

**Table 8:** Processing parameters for line heating in Experiment 2.

Parameters	Start Point	End Point	Power	Velocity of Heat Source	Maximum Processing Temperature
Unit	m	m	W	mm/s	°C
Value	(−0.1, 0.2)	(0.42, 0.2)	43,233	4	800

**Table 9:** Results of various evaluation metrics on the test set.

Metrics	MAE	MAPE	RMSE	R^2^	MedAE	MMaxE	Error Confidence Interval
Unit	°C	%	°C	1	°C	°C	°C
Value	0.5994	0.4622	1.2703	0.9950	0.2511	10.6750	[−0.2031, −0.0627]

**Table 10:** Comparison of computational time for temperature prediction using FEM and the proposed framework in Experiments 1 and 2.

Method	FEM	Proposed Framework	Speedup
Experiment 1	4381 s	71.361 ms	61,392×
Experiment 2	1047 s	19.452 ms	53,824×

## Data Availability

The original contributions presented in this study are included in the article. Further inquiries can be directed to the corresponding author.

## References

[1] Seong W.-J., Jeon Y.-C., Na S.-J. (2013). Ship-Hull Plate Forming of Saddle Shape by Geometrical Approach. J. Mater. Process. Technol..

[2] Liu Q., Li J., Liu J., Lu B., Li H., Chen L. (2025). Hot Deformation Behavior and Constitutive Modeling of High Strength Low-Carbon Alloyed Steel Manufactured by Wire and Arc Additive Manufacturing. J. Mater. Eng. Perform..

[3] Duan J., Farrugia D., Slater C., Li Z., Davis C. (2025). Microstructure Development during Multi-Pass Deformation in a Low Carbon Steel with a Leaner Composition, Finer Grain Size, and Higher Strength. J. Mater. Res. Technol..

[4] Parusov E.V., Chuiko I.M., Gubenko S.I., Oliinyk E.V., Parusov O.V. (2025). Influence of Temperature-Deformation Parameters of Thermomechanical Treatment on the Structure and Mechanical Properties of Low-Carbon Alloyed Steel. Mater. Sci..

[5] Rosenthal D. (1946). The Theory of Moving Sources of Heat and Its Application to Metal Treatments. J. Fluids Eng..

[6] Wang Y., Lu Y., Mendez P.F. (2019). Scaling Expressions of Characteristic Values for a Moving Point Heat Source in Steady State on a Semi-Infinite Solid. Int. J. Heat. Mass. Transf..

[7] Ueda Y., Murakawa H., Rashwan A.M., Okumoto Y., Kamichika R. (1991). Development of Computer Aided Process Planning System for Plate Bending by Line Heating (Report I): Relation between the Final Form of Plate and the Inherent Strain. J. Ship Prod..

[8] Ueda Y., Murakawa H., Rashwan A.M., Okumoto Y., Kamichika R. (1992). Development of Computer Aided Process Planning System for Plate Bending by Line-Heating (Report II): Practice for Plate Bending in Shipyard Viewed from Aspect of Inherent Strain. J. Ship Prod..

[9] Ueda Y., Murakawa H., Rashwan A.M., Neki I., Kamichika R., Ishiyama M., Ogawa J. (1993). Development of Computer Aided Process Planning System for Plate Bending by Line-Heating (Report III): Relation between Heating Condition and Deformation. J. Ship Prod..

[10] Ueda Y., Murakawa H., Rashwan A.M., Kamichika R., Ishiyama M., Ogawa J. (1993). Development of Computer Aided Process Planning System for Plate Bending by Line-Heating (Report IV): Decision Making on Heating Conditions, Location and Direction. J. Ship Prod..

[11] Zhu Y., Luo Y. (2018). Fully Coupled Magneto-Thermo-Structural Analysis by Morphing Method and Its Application to Induction Heating Process for Plate Bending. Int. J. Appl. Electromagn. Mech..

[12] Dong H., Zhao Y., Yuan H., Hu X., Yang Z. (2019). A Simplified Calculation Method of Heat Source Model for Induction Heating. Materials.

[13] Lu Z., Qu J., Liu H., He C., Zhang B., Chen Q. (2021). Surrogate Modeling for Physical Fields of Heat Transfer Processes Based on Physics-Informed Neural Network. CIESC J..

[14] Zhao X., Gong Z., Zhang Y., Yao W., Chen X. (2023). Physics-Informed Convolutional Neural Networks for Temperature Field Prediction of Heat Source Layout without Labeled Data. Eng. Appl. Artif. Intell..

[15] Chen Z., Yang X., Chen N., Yi X., Peng B. (2024). Transient temperature field prediction on anti-icing wing surface based on convolutional temporal networks. Acta Aerodyn. Sin..

[16] Liao S., Xue T., Jeong J., Webster S., Ehmann K., Cao J. (2023). Hybrid Thermal Modeling of Additive Manufacturing Processes Using Physics-Informed Neural Networks for Temperature Prediction and Parameter Identification. Comput. Mech..

[17] Cao X., Duan C., Luo X., Zheng S., Xu H., Hao X., Zhang Z. (2024). Deep Learning-Based Rapid Prediction of Temperature Field and Intelligent Control of Molten Pool during Directed Energy Deposition Process. Addit. Manuf..

[18] Wei Z., Zhao Y., Yuan H., Chang L. (2024). A Physic-Informed Data-Driven Relational Model of Plastic Strain vs. Process Parameters during Integrated Heating and Mechanical Rolling Forming of Hull Plates. J. Mar. Sci. Eng..

[19] Wei Z., Zhao Y., Yuan H., Chang L. (2024). Equivalent Load Model for the Numerical Simulation of Integrated Induction Heating and Mechanical Rolling Forming of Curved Hull Plates. Int. J. Adv. Manuf. Technol..

[20] International Association of Classification Societies (2025). Shipbuilding and Repair Quality Standard.

[21] Zhang L., Reutzel E.W., Michaleris P. (2004). Finite Element Modeling Discretization Requirements for the Laser Forming Process. Int. J. Mech. Sci..

[22] Akiba T., Sano S., Yanase T., Ohta T., Koyama M. (2019). Optuna: A Next-Generation Hyperparameter Optimization Framework. KDD’19: Proceedings of the 25th ACM SIGKDD Conference on Knowledge Discovery and Data Mining, Anchorage, AK, USA, 4–8 August 2019.

[23] Zhao Y., Yuan H., Tang G., Dong H., Hu C., Yan J. (2017). Automatic Integral Forming Method for Double-Curvature Plate of Ship. U.S. Patent.

[24] Zhao Y., Wei Z., Yuan H., Chang L. (2022). An Induction Heating Device. Chinese Patent.

[25] Chen J., Zhu F., Han Y., Xu Z., Chen Q., Ren D. (2022). Global Temperature Reconstruction of Equipment Based on the Local Temperature Image Using TRe-GAN. Appl. Soft Comput..

